# A Computational Framework for Data Fusion in MEMS-Based Cardiac and Respiratory Gating

**DOI:** 10.3390/s19194137

**Published:** 2019-09-24

**Authors:** Mojtaba Jafari Tadi, Eero Lehtonen, Jarmo Teuho, Juho Koskinen, Jussi Schultz, Reetta Siekkinen, Tero Koivisto, Mikko Pänkäälä, Mika Teräs, Riku Klén

**Affiliations:** 1 Department of Future Technologies, University of Turku, 20500 Turku, Finland; 2 Turku PET Centre, Turku University and Turku University Central Hospital, 20500 Turku, Finland; 3 Department of Medical Physics, Turku University Central Hospital, 20500 Turku, Finland; 4 Deparment of Biomedicine, University of Turku, 20500 Turku, Finland

**Keywords:** data fusion, dual gating, MEMS accelerometer and gyroscope, cardiac PET

## Abstract

Dual cardiac and respiratory gating is a well-known technique for motion compensation in nuclear medicine imaging. In this study, we present a new data fusion framework for dual cardiac and respiratory gating based on multidimensional microelectromechanical (MEMS) motion sensors. Our approach aims at robust estimation of the chest vibrations, that is, high-frequency precordial vibrations and low-frequency respiratory movements for prospective gating in positron emission tomography (PET), computed tomography (CT), and radiotherapy. Our sensing modality in the context of this paper is a single dual sensor unit, including accelerometer and gyroscope sensors to measure chest movements in three different orientations. Since accelerometer- and gyroscope-derived respiration signals represent the inclination of the chest, they are similar in morphology and have the same units. Therefore, we use principal component analysis (PCA) to combine them into a single signal. In contrast to this, the accelerometer- and gyroscope-derived cardiac signals correspond to the translational and rotational motions of the chest, and have different waveform characteristics and units. To combine these signals, we use independent component analysis (ICA) in order to obtain the underlying cardiac motion. From this cardiac motion signal, we obtain the systolic and diastolic phases of cardiac cycles by using an adaptive multi-scale peak detector and a short-time autocorrelation function. Three groups of subjects, including healthy controls (n = 7), healthy volunteers (n = 12), and patients with a history of coronary artery disease (n = 19) were studied to establish a quantitative framework for assessing the performance of the presented work in prospective imaging applications. The results of this investigation showed a fairly strong positive correlation (average r = 0.73 to 0.87) between the MEMS-derived (including corresponding PCA fusion) respiration curves and the reference optical camera and respiration belt sensors. Additionally, the mean time offset of MEMS-driven triggers from camera-driven triggers was 0.23 to 0.3 ± 0.15 to 0.17 s. For each cardiac cycle, the feature of the MEMS signals indicating a systolic time interval was identified, and its relation to the total cardiac cycle length was also reported. The findings of this study suggest that the combination of chest angular velocity and accelerations using ICA and PCA can help to develop a robust dual cardiac and respiratory gating solution using only MEMS sensors. Therefore, the methods presented in this paper should help improve predictions of the cardiac and respiratory quiescent phases, particularly with the clinical patients. This study lays the groundwork for future research into clinical PET/CT imaging based on dual inertial sensors.

## 1. Introduction

Intrafraction motion artifacts reduce the image quality and quantitative accuracy of nuclear medicine imaging [1]. In particular, respiratory and cardiac motions may cause image blurring in positron emission tomography (PET), mismatch between computed tomography (CT) and PET images, and CT artifacts, and result in difficulties in delineating boundaries of small targets [2]. In oncological imaging, motion artifacts may result in failure in recognizing small mobile volumes that are potentially cancerous [3]. Respiratory and heart motions may cause underestimation of standardized uptake value (SUV) and variation of lesion volume [4]. Moreover, radiation delivery in the presence of intrafraction organ movement results in a deviation between the intended and delivered dose distributions, which might result in increased organ-at-risk dose and reduced dose in the planned target volume [5]. Therefore, the degradation of image quality due to intrafraction motion and subsequent effects on radiotherapy dose planning and delivery reduce the reliability and accuracy of the clinical interventions, leading to incorrect diagnosis, unnecessary treatment, and insufficient therapy [6]. To minimize motion-related inaccuracies, gating of the acquired PET image by dividing the list-mode data into series of temporal windows which corresponds to the phases of cardiac and/or respiratory phases has shown effective and successful results [6,7,8].

Electrocardiography (ECG) is the most common tool for cardiac gating in PET, while for respiratory gating, different devices such as spirometry, elastic belts with pressure sensors, and optical sensors have been used to track the chest wall or abdomen displacement in both PET and CT [9]. A specific example of the latter methods is the Real-time Position Management (RPM) system by Varian Medical Systems, which uses a camera, a laser, and a marker block, which is attached to the skin of the patient’s chest. The camera then tracks the motion of the marker block, and estimates the respiratory motion based on this. RPM is currently used in clinics for both PET/CT imaging and radiotherapy. However, both the ECG used for cardiac gating and the RPM used for respiratory gating have specific disadvantages—on one hand, ECG presents the instantaneous electrical state of the heart, but does not measure its actual movement; on the other hand, RPM yields only one-dimensional information on the movement of the chest and may fail in reliable tracking of the chest/abdomen due to the breathing irregularities or misplacement of the plastic dotted marker.

Multidimensional motion sensors, namely microelectromechanical systems (MEMS), have been recently proposed for motion correction in clinical imaging. MEMS accelerometers and gyroscopes are miniaturized inertial measurement units which are able to measure upper body micro-movements with six degrees of freedom. Tri-axial accelerometers and gyroscopes measure, respectively, translational and rotational (high-frequency) precordial vibrations originating from the left ventricular contractions and blood flow. Low-frequency longitudinal body movements, induced due to breathing or lungs movements, can be also measured using these sensors. This new method, called MEMS Gating, has shown promising quantitative and qualitative results with the first clinical experiences [10].

A major drawback of using motion sensors in imaging applications is the inevitable intra- and inter-subject variability in the measured signals. To date, several different automated and standalone analysis algorithms have been developed to deal with this challenge [11,12,13,14]. However, it is still essential to develop a multi-functional framework which is able to handle signal motion artifacts—voluntary or involuntary body movements—and accurately estimate fiducial points throughout cardiac and respiration signals. Sensor or data fusion is, for example, a potential approach that can minimize such effects. Methods based on Kalman filters, Bayesian fusion, and other multisensor fusions have been previously introduced, which aim at achieving inherent characteristics of physiological signals not possibly obtainable by a single sensor or source [15]. Although dual MEMS motion sensing with the possibility to concurrently measure both cardiac and respiration signals from multiple orientations is useful, an optimal data fusion approach is essential to fully characterize low- and high-frequency vibration patterns of the lungs and heart motion for gating PET/CT images.

This paper explores a new sensor fusion methodology for motion compensation and gating in PET/CT imaging and radiotherapy. Our approach is based on measuring micro-vibrations in the upper body using highly sensitive MEMS-based inertial measurement units (IMUs), and on fusing this information with that obtained from PET/CT scanners. This paper is a continuation of our earlier contributions [10,16] in which we reported proof-of-concept investigations on MEMS dual gating in PET imaging.

In [16], we considered only accelerometer signals, and no data fusion was used to gather cardiac and respiratory signals. Instead, the signals were visually inspected, and the best-performing axes were manually selected. Continuation of this research was presented in [10], where we also considered gyroscope signals for PET dual gating. In [10], we obtained the cardiac signal by using the envelope signal of the sum of magnitudes of different axes, while the respiration signal was still obtained by manual selection. Even using the envelope signal, the heartbeats were detected from a manually selected axis, which typically is the accelerometer z-axis or the gyroscope y-axis, which cannot be known beforehand due to inter-subject variations in signal morphology. This manual selection was the major drawback of these preliminary studies. To overcome this limitation, we developed an automated way to combine both cardiac and respiratory signals based on independent and principal component analysis, as is described in the following. The new methods described in this paper will be applied to imaging in future work, where we will evaluate the clinical impact of MEMS-based cardiac and respiratory gating.

This paper is organized as follows: In Section 2, we present our signal processing pipeline for cardiac and respiration gating, followed by a brief description on details of clinical data acquisition. In Section 3 we evaluate phase and amplitude characteristics of the obtained signals through the data fusion pipeline and quantitatively assess the performance of the MEMS dual gating, and compare the results to an earlier work. In Section 4 we discuss the presented data processing approach, the potentials and limitations, and describe our future direction in cardiac PET imaging. Finally, Section 5 concludes this work.

## 2. Materials and Methods

This study describes a new data fusion framework for dual cardiac and respiratory gating based on multidimensional MEMS motions sensors. Our approach uses a hybrid fusion based on independent component analysis (ICA) and principal component analysis (PCA) for fusing accelerometric and gyroscopic-derived cardiac and respiration signals from different orientations, respectively. The use of ICA has been previously proposed for noise cancellation in cardiomechanical signals [17]. However, this study presents a novel MEMS-based cardiac and respiratory signal processing pipeline by using ICA and PCA to automate the entire dual gating process for prospective PET/CT and radiotherapy applications.

The accelerometer- and gyroscope-derived cardiac signals correspond to the translational and rotational motions of the chest, and have different waveform characteristics and units. We used fastICA [18] to separate these signals into maximally independent sub-components, and used the first ICA component to represent the underlying cardiac motion. This cardiac pulsation waveform was later used to adaptively detect cardiac systolic and diastolic segments. Additionally, we present and evaluate a novel approach to achieve chest-derived inclination signals (respiratory movements) from the accelerometer and gyroscope sensors. Since accelerometer- and gyroscope-derived respiration signals represent the inclination of the chest, they are similar in morphology and have the same units. Therefore, we use principal component analysis (PCA) to combine them into a single signal. The first component of PCA—the one with the largest variance—is used to represent the overall inclination signal.

### 2.1. Data Acquisition and Experimental Protocol

**Figure 1:**
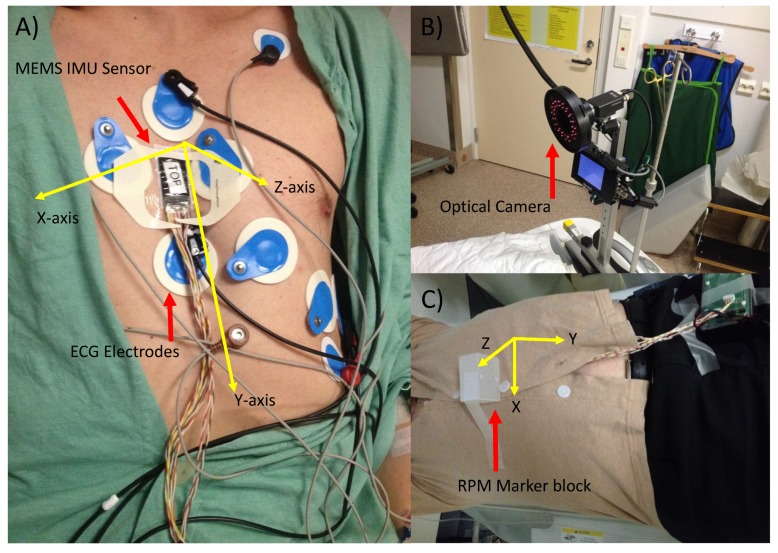
Simultaneous recording of electrocardiography (ECG), chest acceleration and angular velocity (**A**); and real-time position management (RPM) (**B**). The eight blue electrodes are for ECG, while the rectangular black sensor is the inertial measurement unit containing the accelerometer and the gyroscope. Yellow-coloured arrows show the axes of motion in three different orientations, the x- and y-axes point left-to-right and head-to-foot, while the z-axis points from the dorsal to the ventral side, respectively. With the RPM camera, only longitudinal chest/marker-block movement in the z-axis was obtained (**C**).

MEMS data were collected using a previously designed inertial sensor array [10]. A triple-axis capacitive digital accelerometer (Freescale Semiconductor, MMA8451Q, Austin, TX, USA) and a three-axis angular rate (gyroscope) sensor (Maxim Integrated, MAX21000, San Jose, CA, USA) was used. This joint embedded sensor array was configured to function as a six-degrees-of-freedom inertial measurement unit (IMU) in order to measure the cardiogenic and respiration motions of the upper chest. A two-lead electrocardiogram signal (ADS1293 from Texas Instruments, Dallas, TX, USA) was used as a reference signal. A reference optical camera with real-time position management (Varian, Palo Alto, Santa Clara, CA, USA) was used as a reference respiration sensor. All measurements were analyzed offline, and the recorded physiological signals were processed using Matlab (version 2015). Accelerometer, gyroscope, and ECG signals were recorded simultaneously with a sampling frequency $\left(F_{s}\right)$ of 800 Hz; RPM was recorded with a sampling rate of 25 Hz. All MEMS data were recorded with the sensors attached to the body of sternum using a double-sided tape. From our experience, this anatomical location provides signals of the highest quality and does not significantly suffer from operator dependency on the sensor placement. The RPM marker was attached to either the chest or the abdomen of the subjects. The subjects were lying in the supine position. The axes of rotations/translations were defined as follows: the x-axis points were oriented laterally from left to right (sinister–dexter), the y-axis points were directed from head to foot (superior–inferior), and the z-axis points were oriented from the back to the chest (dorsoventral). Figure 1 shows an overall view of the measurement configuration including the MEMS sensor unit, ECG, and RPM modalities.

Seven healthy adult subjects (7 male) and 19 patients with a history of coronary artery disease (CAD; 15 males and 4 females) participated in the experiments of this study. The dataset (DS) for the healthy group (DS I) consisted of about 420 min of synchronized, accelerometer, gyroscope, ECG, and RPM recordings in total. The dataset for the patient group (DS II) consisted of about 17,280 min of accelerometer, gyroscope, ECG, and optical camera (RPM) recordings in total. The measurements were performed at Turku PET Centre, and the study was conducted in accordance with the Declaration of Helsinki. The clinical study protocol was approved by the Ethical Committee of the Hospital District of Southwestern Finland (ETMK 44/180/2012). A third group (DS III) of subjects, including 12 young healthy volunteers was also considered in this study to further validate the methodologies presented in this paper. Table 1 describes a summary of the demographic information for these three study groups, including the min–max, mean, and standard deviation (SD) of their age, height, weight, and body mass index (BMI).

### 2.2. MEMS-Based Chest Motion Processing

#### 2.2.1. Rotational and Translational Cardiac Motion Fusion

Cardiogenic vibration signals derived from multi-axial accelerometer and gyroscope sensors were processed for prospective cardiac gating in this study. To this end, the first step was to detect heartbeats from the precordial signals whose morphological characteristics may relatively indicate mechanical functioning of the heart [10,19]. In order to achieve this goal, two major issues, mainly dealing with the processing of the MEMS-derived cardiac signals, needed to be considered. First, unlike the ECG signal, we here dealt with cardiac acceleration and angular velocity signals, each representing specific characteristics of the heart functioning in three different orientations. For practical gating purposes, it is imperative to properly select the best-performing axes/channels before trying to detect cardiac quiescent phases. The second problem is considerable inter- and intra-subject variability with the MEMS-derived signals which may hamper robust processing of these signals. To deal with these challenges, we previously developed an algorithm based on the Hilbert transform for detecting heartbeats and segmenting cardiac quiescent phases. Although this algorithm seemed to work reliably for the healthy subjects, it still suffered from certain limitations, such as robustness in detection of irregular morphology, amplitude, and clinically relevant features of the signal in the heart-diseased subjects. One potential solution to tackle these two challenges is data fusion using ICA.

Independent component analysis found a linear representation of non-Gaussian data so that the components were statistically independent [20]. Assuming that precordial heart movements were generally constituted from rotational and translational components, we denoted the measured signals by Rotation(t) and Translation(t). For the sake of simplicity, we assumed that each of these motion signals was a weighted mixture (sum) of precordial vibrations from two hidden underlying components (e.g., myocardial movement and ballistic forces due to the blood flow), which we denoted by $S_{m}\left(t\right)$ and $S_{b}\left(t\right)$. We can show this angular and translational vibration mixture as a linear equation:

(1) $$\begin{array}{c} Rotation\left(t\right)=a_{11}S_{m}+a_{12}S_{b}\\ Translation\left(t\right)=a_{21}S_{m}+a_{22}S_{b} \end{array}$$

where $a_{11}$, $a_{12}$, $a_{21}$, and $a_{22}$ are the unknown coefficients or mixing weights that define the representation of the observed signals. The original underlying signals ($S_{m}$ and $S_{b}$) can be recovered by multiplying the observed signals (Rotation(t) and Translation(t)) with the inverse of the mixing weights ($a_{ij}$). In practice, ICA, as a generative model, helps estimate the $a_{ij}$ based on the information of their independence, which allows us to recover the original signals. The ICA model describes how the observed data are generated by a process of mixing the components, here which are $S_{m}$ and $S_{b}$.

**Figure 2:**
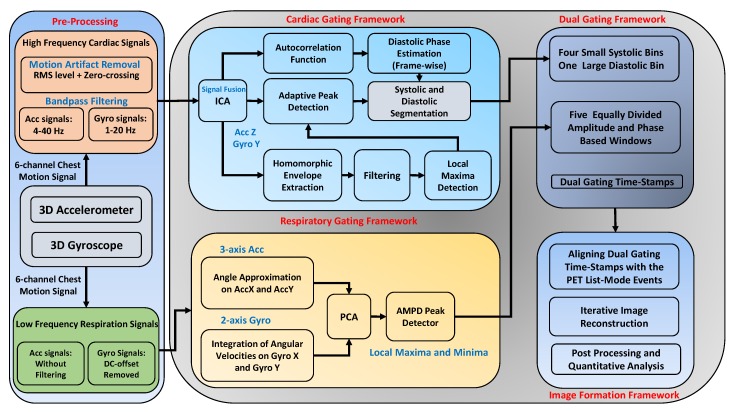
Microelectromechanical (MEMS)-based cardiac and respiratory gating framework using joint rotational and translational motion sensing.

To start with ICA, a primary motion artifact removal is first used to eliminate sporadic waveforms due to noise/artifacts. This motion artifact removal includes a fast frame-wise (500 ms) estimation of the signal’s root mean square (RMS) where the signal frames having an RMS greater than three times the median value of the RMS level are found. From these frames, the beginning and ending position of the noisy/sporadic spikes are defined, and the middle points are replaced by zeros (zero-padding). Afterwards, a band-pass filter was applied to remove high-frequency signal content associated with Gaussian noise and respiratory baseline wandering (dc offset) from the accelerometer (4–40 Hz) and gyroscope (1–20 Hz) signals. Figure 2 shows a general overview of the proposed dual cardiac and respiratory gating framework based on only MEMS motion sensors.

Following the signal preprocessing, an ICA was adapted to combine translational and rotational signals. Having a multi-axial sensor unit, previous research established that heart accelerations in dorsoventral and angular velocities in superior–inferior orientations display a specific repeating pattern characterized by several peaks and valleys reflecting specific events of the beating heart [21,22]. Therefore, accelerations measured from the z-axis, and angular velocities from the y-axis were fed into an ICA function in order to achieve independent components estimating the overall chest vibration, while rejecting noise/interference components from these signals. Angular velocities measured from the z-axis were not included here, as there were no substantial cardiogenic micro-rotations in the dorso-ventral axis, as highlighted in our previous investigation [22]. Prior to ICA, the range of the motion signals was standardized using a zero-mean normalization. After this ICA operation, the first IC which had the maximum possible information was picked from each set of data. The resulting signal (corresponding to the first independent component) was deployed for cardiac gating, as described below. In the following, we describe a fully automated waveform delineation and cardiac quiescent phase segmentation using a standalone heartbeat detection pipeline. Heartbeat detection and cardiac cycle segmentation is essentially needed in order to accurately align cardiac and respiratory quiescent periods.

#### 2.2.2. Standalone Cardiac Cycle Segmentation

After the ICA, we applied a previously developed wavelet enhancement filter [23] to obtain a robust estimation of the location of cardiac mechanical impulses. The reason to have this envelope-shaped signal was because our previously described adaptive peak detection process [24] required two input signals to accurately estimate the position of the heartbeats or delineate the onset of the systolic phases. The resulting envelope derived from the MEMS cardiac signal (corresponding to the first independent component) was deployed to estimate the position of the heartbeats using an automatic multi-scale peak detection (AMPD) technique given by [25]. These local maxima (heartbeats) found from the homomorphic pulsatile signal together with the original ICA-derived cardiac signal were subsequently fed to another adaptive peak detection function, as described in [24]. This adaptive heartbeat detector utilized a refinement process in which a search window was defined to relocate the closest heartbeat candidate in the ICA-derived cardiac signal to the peak which was already detected throughout the homomorphic envelope by the AMPD method. This refinement process was updated upon the noise and signal level thresholds, as described by [26].

Following this refinement process, we applied a primary cardiac cycle segmentation function to estimate sequential phases of cardiac cycle, mainly the ejection time, by adapting an autocorrelation analysis. To this end, we considered a non-overlapping segment-wise (20-s) short-time autocorrelation, with a maximum 5 s lag, in order to identify harmonic frequencies. In practice, the heart generates a series of vibrations at the time of a systole (cardiac contraction) which, by repeating, constitutes the periodicity of the signal, or the heart rate. Another set of diastolic precordial vibrations appear about 300–400 ms after the first contraction (end-systole), which typically pose weaker amplitudes and happen very quickly (sharp spikes). These diastolic vibrations also have a periodic pattern which can be identified by the autocorrelation analysis.

**Figure 3:**
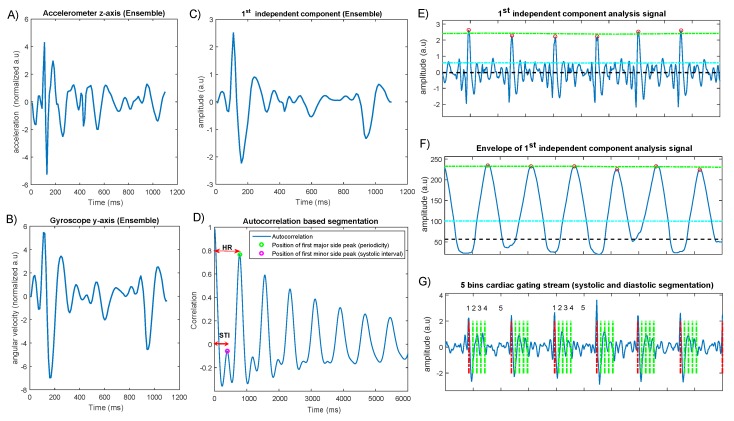
Cardiac gating process based on dual accelerometer and gyroscope sensing. Panels (**A**,**B**) show the ensemble average of $Acc_{z}$ and $Gyro_{y}$ signals obtained from the inertial measurement unit (IMU) signals; Panel (**C**) is the ensemble average of the first independent component after applying the independent component analysis (ICA) function; and Panel (**D**) is the corresponding autocorrelation which was applied to estimate the systolic and diastolic phases; Panel (**E**) shows the adaptive heartbeat detection of the fused signal with the help of the envelope signal shown in Panel (**F**); and Panel (**G**) is the final segmented signal with the information obtained from Panel D, showing four small consecutive systolic bins and one large diastolic bin to be used in cardiac gating.

The short-term autocorrelation function returns multiple side-peaks if the signal is periodic, and by default, the distance from the first dominant side-peak to the largest spike occurring at zero lag determines the cardiac cycle periodicity. Once the first side-peak is detected, a secondary search-back function is used to pinpoint the largest semi side-peak within the distance between the zero lag and the first side-peak. As a result of this frame-wise autocorrelation process, the estimated time interval from this semi side-peak will correspond to the ejection time. This rough estimation of the systolic phase is based on an assumption that the ejection time—the time between the aortic valve opening and closure—does not considerably change within the 20-s frames/windows (see Figure 3D).

This segmentation process is followed by another step in which the cardiac cycle can be divided into *n* bins/windows, where n=5 in the example shown in Figure 3G). In cardiac gating, the first n-1 bins equally divide the systolic phase (corresponding to left ventricular ejection period), while the $n^{th}$ bin comprises the entire diastolic phases. Optimally, by defining the end-diastolic phase (here, bin number 5) as the largest bin, a large number of PET event counts can be saved in one bin where the cardiac motion is also minimal. This allows a better differentiation of small targets in PET imaging. For example, when minimizing the cardiac motion in coronary artery imaging of the vulnerable plaque in PET, it is desired to define a large gating bin based on the end-diastolic phase of the cardiac function, as suggested in [27,28]. This sequential temporal segmentation of the cardiac cycles is in line with the respiratory derived bins, all of which should be aligned with the list-mode data.

Figure 3 illustrates step-wise data fusion for the tri-axial accelerometer and gyroscope signals and complementary autocorrelation function for cardiac cycle segmentation.

#### 2.2.3. Decomposition of Chest Angular Movements

For the purpose of respiratory gating using MEMS sensors in cardiac PET/CT, we previously described a moving-average filter to obtain accelerometric- and gyroscopic-derived respiration signals, denoted by ADR and GDR, and through a segmentation process for which we estimated quiescent respiratory periods suitable for amplitude- or phased-based respiratory gating [10]. In this study, low-frequency chest motion components were extracted from the precordial high-frequency movements using an updated respiration signal extractor (RSE) algorithm, as described in the following.

**Figure 4:**
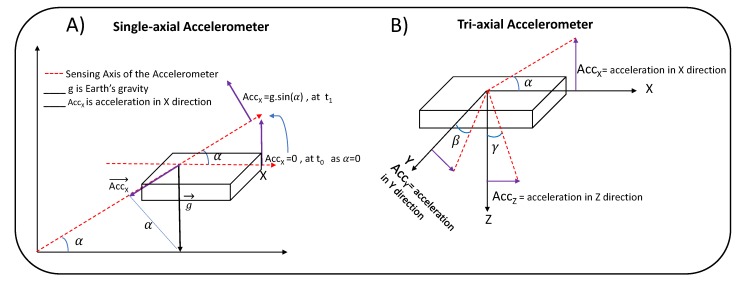
Angle measurement using a single (**A**) and multidimensional (**B**) accelerometer sensor for tracking respiratory-induced chest inclination.

The RSE algorithm presented in the context of this paper decomposes low-frequency components, namely chest inclination/rotation, from the raw accelerometer and gyroscope signals. The breathing led to a periodical and low-frequency movement of the thorax, and thus it changed the inclination of the accelerometer placed on the chest. With the gyroscopic signals, an integration process, which also acts as low-pass filtering, was used in order to derive chest inclinations or the angle of rotation (tilt) from the x- and y-axis. Similarly, chest inclinations—the pitch around the x-axis and roll around the y-axis—could be obtained from the accelerometers by calculating angular chest displacements, as shown in Figure 4. Note that, unlike gyroscopes, accelerometers are not able to measure yaw, which is rotation around the z-axis.

Figure 4A illustrates a single axis accelerometer sensor measuring angular displacement around its y-axis, here denoted α, as acceleration $Acc_{x}$ in this direction depends on gravity changes, along with angular displacement. Using the same trigonometry and a multidimensional sensor, we calculated inclination around the x-axis (pitch) on the basis of dividing accelerations in the y-direction over the total magnitude of gravity in the direction of the z- and x-axes, while the angular position of the sensor altered with β (see Figure 4B). Therefore, the chest pitch angle can be computed as

(2) $$\begin{array}{c} Pitch\;or\;\beta =\arctan(\frac{Acc_{y}}{\sqrt{Acc_{z}^{2}+Acc_{x}^{2}}}). \end{array}$$

Notice that since the z-axis contains gravitational accelerations whose magnitude is considerably bigger than the precordial accelerations in the x- and y-axis directions ($Acc_{z}$≈ 9.8 m/s${}^{2}$), one can approximate β as

(3) $$\begin{array}{c} \beta \approx \arctan\left(\frac{Acc_{y}}{Acc_{z}}\right)\Rightarrow \beta \approx \frac{Acc_{y}}{Acc_{z}}\approx \frac{Acc_{y}}{g}. \end{array}$$

Likewise, rotation around the y-axis (roll) depends on the total magnitude of gravity with the x- and z-axes, while the accelerometer tilts with an angle of α

(4) $$\begin{array}{c} Roll\;or\;\alpha =\arctan\left(\frac{Acc_{x}}{\sqrt{Acc_{z}^{2}+Acc_{y}^{2}}}\right)\approx \arctan\left(\frac{Acc_{x}}{Acc_{z}}\right)\Rightarrow \alpha \approx \frac{Acc_{x}}{Acc_{z}}\approx \frac{Acc_{x}}{g}. \end{array}$$

A previously designed narrow band-pass filter with the frequency band [0.1–2 Hz] was subsequently deployed to discard high-frequency components from the angular displacement signals.

**Figure 5:**
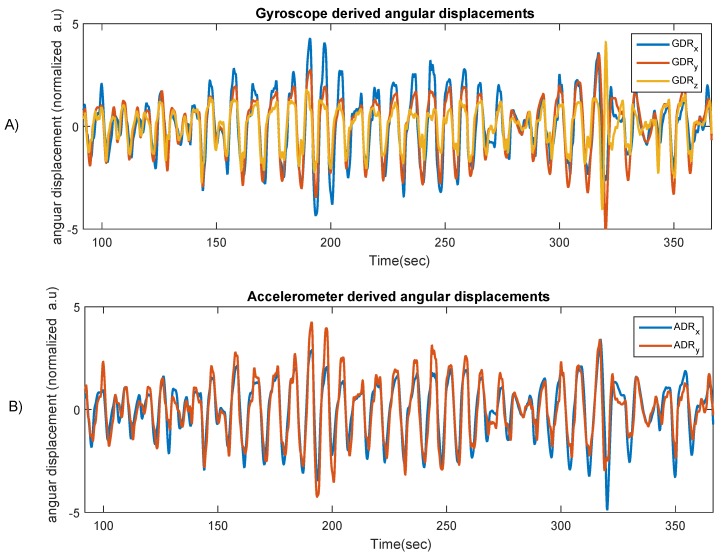
Gyroscope (**A**) and accelerometer-derived (**B**) chest angular displacements. (**A**) Tri-axial chest’s angular displacement obtained from gyroscope; (**B**) angular displacements obtained by accelerometer from the x and y directions (chest inclination).

Figure 5 shows the chest angular displacement curves derived from gyroscope sensors (all three axes) and accelerometer in the x and y directions. As shown in this figure, there is no significant difference between the ADRs and GDRs, as a near-perfect association was visible with both MEMS-derived signals and the reference optical RPM-derived respiration signal.

#### 2.2.4. Chest-Derived Respiratory Motion Fusion

**Figure 6:**
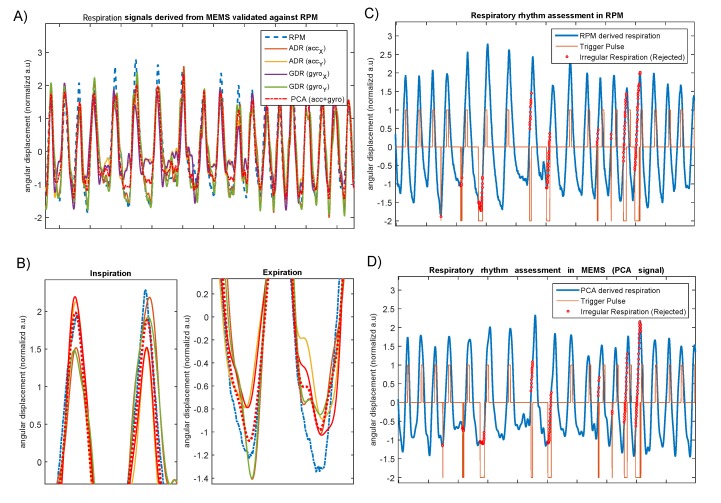
RPM and MEMS-derived chest longitudinal displacements. Chest’s angular displacement obtained from accelerometer and gyroscope sensors in x and y directions (**A**,**B**) and chest angular displacement obtained by RPM (**C**) and principal component analysis (PCA) from the five-axis MEMS data (**D**). The red-colored pulse wave and corresponding dots represent the signal quality and places where the RPM system rejected breathing flow.

The use of multi-axial MEMS sensors can help in the estimation of chest movements in different orientations. However, in order to achieve the optimal performance of the multi-axial motion sensing, it is advantageous to apply proper techniques which may yield a better estimation of the lung and upper chest movements. For example, simple averaging of these signals may not necessarily yield a robust estimation of the chest inclinations, as summing the signals element-wise can either yield a constructive or a destructive effect. Therefore, we applied the principal component analysis function in order to combine both the ADR and GDR signals. Principal component analysis is one of the oldest and most popular techniques for dimensionality reduction by linearly mapping data to a lower dimensional space while retaining the maximal amount of variance [29]. PCA converts a series of observations of interrelated variables (here, respiration waveforms) into a series of values of linearly uncorrelated variables called principal components, which are ordered so that the first contains the most variance of the original variables. In this study, we propose orthogonal transformation of the data from multiple axes of two MEMS sensors to improve the overall estimation of the chest’s low-frequency vibrational movements. The reason behind this PCA-based respiratory data fusion is, again, inter-subject and inter-axis variability, in which the morphology and amplitude of the respiration signals derived from the x and y axes may differ patient by patient. Additionally, we only needed one single respiratory signal for the gating process, and therefore it is essential to choose the best-performing signal for this purpose. Hence, we combined the ADR${}_{x}$, ADR${}_{y}$, GDR${}_{x}$, GDR${}_{y}$, and GDR${}_{z}$ signals to achieve a better, but also robust estimation of the chest’s cyclic movements. For the sake of simplicity, we stacked respiration vector data into a matrix (*R*), as

(5) $$R={\left[ADR_{x}\;\;\;ADR_{y}\;\;\;GDR_{x}\;\;\;GDR_{y}\;\;\;GDR_{z}\right]}^{T},$$

where indices *x*, *y*, and *z* represent the orientation of the chest displacements measured by the accelerometer and gyroscope. With this setting, *R* was considered as the measurement matrix for the PCA data fusion, and with that, we aimed at transforming data obtained by two sensors from two different orientations without discarding any motion information. Figure 6 shows the results of the data fusion where the PCA-derived respiration signal follows the reference optical sensor, as well as ADR and GDR.

In order to further investigate the reliability and reproducibility of the MEMS-derived signals, we performed a primary correlation analysis, where the results are shown in the following section. Additionally, we measured the mean absolute trigger offset by calculating the time differences between the trigger points—peak inhalation phases (local maxima)—in RPM and PCA traces [30]. However, before computing the correlations, one needs to carefully recognize the polarity of the obtained respiration signals. This is critical, as for the phase-based gating the location of peak inhalation and exhalation points can help estimate the respiratory quiescent phases. To this end, we initially compared the correlation of the respiration signals in x and y directions against the chest longitudinal movements in the z direction as a reference polarity. Those axes with a negative correlation coefficient were simply inverted to turn the signal to its correct polarity.

## 3. Results

### 3.1. Validation of Respiratory and Cardiac Data

We performed a quantitative assessment of the accuracy of the respiratory waveform measured by the gyroscope, accelerometer, and their combination as compared to a traditional technique for respiratory monitoring, such as the respiration belt and real-time position management (Varian’s RPM) which were taken as the gold standard.

#### 3.1.1. ADR and GDR against Optical Camera (RPM)

Table 2 and Table 3 provide the inter-correlations between the MEMS-derived respiration signals, as well as their corresponding PCA-derived signal against the reference optical sensor, RPM. The results obtained from the preliminary analysis of correlations show a significant Pearson correlation coefficient (*r*). With the PCA, the average correlation coefficients were 0.87 ± 0.08 (*p*< 0.001) and 0.73 ± 0.17 (*p*< 0.001) for the healthy and diseased groups, respectively, indicating a fairly strong positive correlation against RPM. Similarly, the average correlation coefficient ranges between the other respiration signals—derived from different orientations—and RPM were 0.63 to 0.69 and 0.46 to 0.52. On average, the absolute time offset between PCA-driven and RPM-driven triggers were 0.23 ± 0.15 s (range: 0.12 to 0.37 s) for the healthy group and 0.31 ± 0.17 (range: 0.12–0.37 s) for the diseased group.

An important finding is that correlation coefficients obtained from one axis in either the gyroscope or accelerometer was slightly different to the other axis, showing that inclination characteristics of one single axis, in either x, y, or z orientations, is not necessarily consistent and may vary subject by subject. However, PCA-derived respiration still remains to have a coherent estimation of the total chest inclination, resulting in a robust prospective respiratory gating.

**Figure 7:**
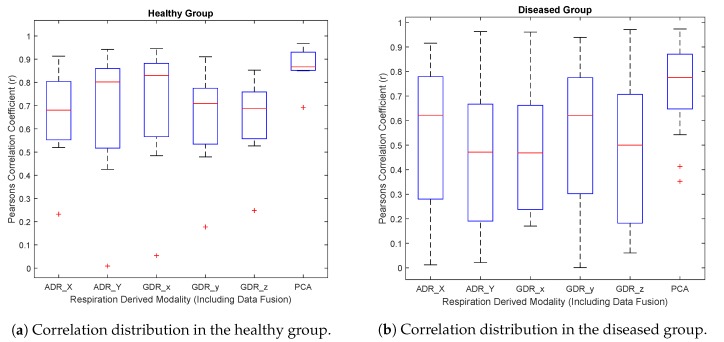
Box plot of the correlation coefficients between RPM and MEMS-derived respiration signals.

Figure 7a,b present the distribution of correlation coefficients among the 7 healthy and 19 coronary artery-diseased subjects. This result is significant at the *p*-value < 0.001. As can be seen, in both groups, PCA shows the highest median level and a fairly narrow interquartile range (IQR) as compared to the GDR and ADR correlation coefficients. From the box plots in this figure, it is also apparent that the PCA-derived respiration shares the highest similarity with the optical sensor.

As can be noted, the average correlations with the diseased group tend to be lower than with the healthy group. This can be explained by several factors. First, only seven young study subjects in the healthy group were considered, and it was assumed that they had a fairly steady breathing rate. They were asked to remain silent and motionless, and they were fully cooperative. With the patient group, we considered 19 subjects with fairly larger breathing rate variations, and they did not fully follow the study guidelines due to many reasons. However, as Figure 7 shows, the median PCA-derived correlation coefficients obtained for the healthy and diseased groups were still very close and fairly high. Overall, these results suggest that there is an essential need to have a reliable data-fusion approach in order to have a solid estimation of chest motion. The results of the correlation analysis supports the fact that PCA-derived respiration from MEMS multi-axial sensors can be reliably used for amplitude and phase-based respiratory gating.

#### 3.1.2. Beat-to-Beat Estimation of the Cardiac Cycles and Related Cardiac Events

The detection performance of the presented method in beat-to-beat estimation of heartbeats was evaluated and computed separately for each subject using different metrics (as described previously in [24]). We considered only diseased subjects, as this group tended to have the most challenges in cardiac signals for the peak detection task. The results are shown in Table 4. As can be seen, the algorithm performance was compared against the previously developed Hilbert algorithm [10,24]. The same dataset (DS II) was used for both algorithms, whilst the Hilbert algorithm deployed only an accelerometer z-axis or gyroscope y-axis. With the ICA-based signal fusion, the average true predictive rate (TPR), positive predictive value (PPV), and F1-measure values in this study were 0.94 ± 0.06, 0.93 ± 0.08, and 0.93 ± 0.06, respectively, and these rates showed better performance compared to the earlier contribution. Additionally, the root mean square error (RMSE) was 58.4 ± 37.6 ms, showing an almost perfect detection performance for the ICA signal fusion as compared to those obtained by single-axis accelerometer (z-axis) and gyroscope (y-axis) signals. It is worth noting that the Hilbert method relies solely on the signal quality of the manually selected axis, which is not ideal for automated beat-to-beat analysis of the cardiac motion signals.

The average HR estimations for the healthy group were 60.2 and 59.4 for the ECG and MEMS, respectively. With the diseased group, the average heart rates for the ECG and ICA were 74.8 and 74.26 beats per minute (bpm), respectively. In addition to the heart rates, we calculated the cardiac cycle percentage (CCP) by dividing the estimated beat-to-beat total systolic intervals in the ICA signal to the total length of the corresponding cardiac cycles. Accordingly, the average total systolic time interval and CCP for the healthy group were 343 ms and 29%, respectively. On the other hand, with the diseased group, the average systolic time interval and CCP were 511 ms and 54%, respectively. This implies that the mean estimated cardiac relaxation phase in the diseased group was fairly shorter (about 45%) as compared to the healthy group with an average estimated diastolic quiescent phase of 70%.

#### 3.1.3. Estimation of the Breathing Cycles (Peak Inhalation and Exhalation)

We also used another automated peak detection algorithm to estimate the respiration cycles. This algorithm is based on the consolidation of local maxima and minima points in order to measure the amplitude and phase variations within the respiration signal. The method first estimates the location of the local minima and maxima points using the AMPD algorithm from each axis, and following that, computes the median distance between the peak candidates (in both inhalation and exhalation regions) to roughly estimate the correct location of the peak inhalation and exhalation in the surrogate signal. Depending on the desired gating method for the image reconstruction in PET/CT imaging, the amplitude and phase ranges of the patient’s breathing signal can be divided evenly into *n* bins. An interesting future research topic is to compare quantitatively the accuracy of target segmentation in amplitude-based and phase-based respiratory gating schemes.

### 3.2. Respiration Belt

**Figure 8:**
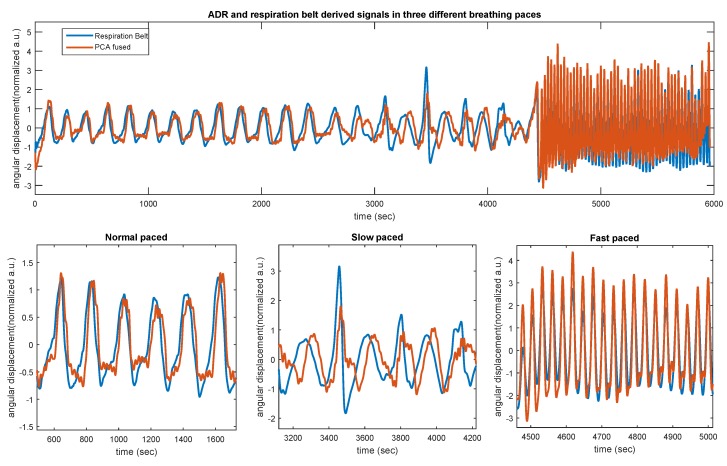
Respiration belt against ADR${}_{x}$ and ADR${}_{y}$ chest rotational movements and corresponding PCA signal during three different breathing patterns. All signals have been scaled to zero mean to match the respiration belt signal to ease the comparison.

To further assess the performance of the PCA-derived respiration, the breathing curves were also extracted from a single tri-axial accelerometer and a piezoelectric respiratory belt transducer (AD Instruments, MLT1132, Dunedin, New Zealand), which were recorded simultaneously. While this measurement device did not contain a gyroscope, it provided us with a possibility to investigate how the presented MEMS respiratory gating works with accelerometer only. To obtain such an assessment, we considered 12 young, healthy subjects from our previous study [16] to obtain respiration signals in three breathing conditions (normal, slow, and fast-paced breathing). Subjects were asked to control their breathing with each pace. Table 5 shows Pearson correlations between ADR x and y against the respiration belt curves. As shown, and similarly to the previously described results, PCA-derived respiration shows fairly better correlation coefficients, though moderate, during normal (0.51 ± 0.22; *p* < 0.01) and slow (0.55 ± 0.11; *p*< 0.01)-paced breathing. However, during the fast-paced breathing, the average correlation coefficient was relatively higher, 0.74 ± 0.12; *p*< 0.01, which indicated a good linear relationship with a statistically significant association. There are two possible explanations for these results. One possible explanation for the moderate inter-correlations during normal and slow-paced breathing might be the lack of gyroscope information. Another possible explanation for this is that typical piezoelectric respiration belts are less sensitive to the very low-frequency chest inclinations, leading to a weaker inter-correlation during slow-paced breathing. However, the belt and MEMS have shown a fairly strong correlation during the fast-paced breathing, covering this hypothesis. As shown in Figure 8, in all three breathing sessions, PCA has shown fairly better inter-correlation to the respiration belt, which is in line with the results obtained in the previous sections.

## 4. Discussion

In this study, a sensor/data fusion framework was presented to combine accelerometer- and gyroscope-derived cardiac and respiration signals. PCA and ICA are well-known methods which are based on statistical transformation of multidimensional data. The ICA-based estimated cardiac signal—originating from precordial vibrations on the surface of the chest wall—is used for cardiac gating and predicting quiescent phases of the heart’s motion. Additionally, we proposed a novel method to estimate angular displacement of the chest obtained by the accelerometer and gyroscope sensors. Gyroscopic-derived angular displacement signals, together with accelerometric-derived chest inclinations can provide a robust estimation of the respiratory chest movements. We also combined a respiratory chest motion by taking the PCA of the obtained respiration signals from the two MEMS sensors to be used for respiratory gating in PET. Having combined cardiac and respiratory signals, we performed a validation study with healthy and diseased groups of volunteers.

As can be seen from Figure 7, the correlations between RPM and MEMS signals vary between the healthy and the diseased group. This may not only be due to the clinical status, but also due to the demographic differences, such as the age distribution, of these groups. Another cause of inter-subject variation in the MEMS signals may be the BMI, and while we included persons with high BMI in both the healthy and the diseased group (as shown in Table 1), the actual effect of obesity needs to be investigated with a larger group of subjects. However, in our preliminary study [10] we did not see signal quality loss with obese healthy subjects.

The use of multidimensional MEMS motion sensing is advantageous. Dual gating using MEMS inertial sensors is a low-cost solution (as compared to both RPM camera and ECG devices), and is a one-step process where both the cardiac and respiratory motion can simultaneously be achieved using a single device with easy set-up. Naturally, the device offers an inherent advantage in motion tracking, allowing us to trace the mechanical movements of the lungs and the heart motions as opposed to using an optical external signal with an assumed correlation with internal motion (RPM) and the electrical function of the heart (ECG). In fact, multi-axial MEMS motion sensors measure precordial and respiratory mechanical movements, allowing for selection/transformation of the motion signals in terms of signal morphology and waveform stability for both cardiac and respiratory gating.

Data fusion techniques presented in this study revealed a reliable combination of MEMS motion signals that can lead to a robust estimation of cardiac and respiration motion. The method is also compatible for the clinical PET studies, and may improve PET instrumentation logistics as only a single miniaturized and mountable IMU sensor can be used to track intrafraction, including both respiratory and cardiac motions, without any supplementary equipment. An interesting future research topic is how to investigate the development of a hybrid gating approach, including electrical, optical, and mechanical sensing modalities that can potentially be applied in clinical cardiac and/or oncological studies, leading to an enhanced diagnostic quality, improved patient safety and comfort, fewer logistic complications, and less radiation doses. Yet, continued efforts are needed to make such a system more accessible to the clinical routines.

The most important next step in this research is to apply the data fusion approach in the gating of PET/CT images in a clinical setting. This will allow us to perform a quantitative evaluation of the MEMS-based dual gating. Furthermore, our presented approach based on linear data transformation techniques was tested in an offline processing scheme on the entire set of recorded signals, while for the real-time considerations, one needs to obtain corresponding ICA/PCA coefficients prior to the target dual gating or radiotherapy. Our future task is, therefore, applying this framework in real time, along with appropriate clinical assessments.

## 5. Conclusions

The purpose of the current study was to determine a new data fusion framework to simplify dual cardiac and respiratory gating using MEMS sensors. The research has shown that ICA and PCA techniques can relatively minimize the complexity of the MEMS gating process, allowing for maximum deployment of cardiomechanical and chest rotation information obtained from two multi-axial motion sensors. Overall, this study strengthens the idea that MEMS sensors can provide reliable and reproducible information regarding the characteristics of the intrafraction motions generated by the body’s internal organs, such as the heart and lungs. Further research could also be conducted to determine the effectiveness of MEMS gating in clinical PET/CT.

## Figures and Tables

**Table 1:** Demographic information of the study subjects.

Age (Year)			Height (cm)		Weight (kg)		BMI (kg/m${}^{2}$)	
**Dataset**	**Min–Max**	**Mean**±**SD**	**Min–Max**	**Mean**±**SD**	**Min–Max**	**Mean**±**SD**	**Min–Max**	**Mean**±**SD**
DS I	27–39	32 ± 4.0	172–190	178 ± 6.0	60–120	88 ± 19.0	20–34.0	27.0 ± 5.0
DS II	44–84	61 ± 10.0	153–200	176 ± 11.0	47–116	88 ± 17.0	22.4–32.5	28.8 ± 3.4
DS III	23–38	28 ± 4.8	173–190	179 ± 5.0	65–85	75 ± 7.7	20.7–25.7	23.2 ± 1.5

**Table 2:** Pearson’s correlation coefficients and trigger time offset between RPM and PCA traces. Triggers were generated at peak inhalation (local maxima of respiratory cycles) phases and compared for RPM triggers.

Healthy Group Correlation)							Mean Trigger Offset (s)
**Subject**	**ADRy**	**ADRx**	**GDRy**	**GDRx**	**GDRz**	**PCA**	**|RPM-PCA|**
1	0.65	0.42	0.48	0.70	0.65	0.69	0.29 ± 0.17
2	0.52	0.79	0.87	0.47	0.52	0.86	0.20 ± 0.17
3	0.83	0.94	0.94	0.79	0.85	0.96	0.18 ± 0.13
4	0.91	0.82	0.82	0.91	0.73	0.93	0.12 ± 0.08
5	0.72	0.05	0.05	0.69	0.24	0.85	0.37 ± 0.19
6	0.68	0.80	0.81	0.72	0.76	0.85	0.20 ± 0.15
7	0.23	0.87	0.88	0.17	0.69	0.91	0.22 ± 0.16
average	0.65	0.66	0.69	0.64	0.63	0.87	0.23
std	0.20	0.31	0.30	0.22	0.18	0.08	0.15

**Table 3:** Pearson’s correlation coefficients and trigger time offset between RPM and PCA traces within the diseased subjects.

Diseased Group (Correlation)							Mean Trigger Offset (s)
**Subject**	**ADRy**	**ADRx**	**GDRy**	**GDRx**	**GDRz**	**PCA**	**|RPM-PCA|**
1	0.80	0.17	0.17	0.80	0.51	0.67	0.34 ± 0.20
2	0.92	0.76	0.79	0.94	0.81	0.91	0.28 ± 0.18
3	0.85	0.92	0.92	0.84	0.25	0.93	0.16 ± 0.19
4	0.65	0.55	0.48	0.44	0.84	0.81	0.33 ± 0.10
5	0.87	0.55	0.53	0.90	0.88	0.85	0.24 ± 0.20
6	0.08	0.22	0.23	0.07	0.06	0.41	0.27 ± 0.18
7	0.91	0.96	0.96	0.92	0.97	0.97	0.19 ± 0.13
8	0.08	0.16	0.17	0.09	0.06	0.69	0.23 ± 0.17
9	0.38	0.37	0.37	0.36	0.32	0.89	0.29 ± 0.16
10	0.56	0.02	0.26	0.69	0.58	0.88	0.33 ± 0.18
11	0.69	0.53	0.61	0.62	0.68	0.81	0.39 ± 0.19
12	0.28	0.74	0.74	0.56	0.72	0.78	0.30 ± 0.18
13	0.62	0.14	0.17	0.64	0.60	0.64	0.27 ± 0.18
14	0.33	0.18	0.19	0.34	0.13	0.35	0.47 ± 0.17
15	0.29	0.68	0.68	0.29	0.49	0.67	0.22 ± 0.14
16	0.72	0.63	0.61	0.71	0.33	0.79	0.18 ± 0.14
17	0.69	0.35	0.37	0.68	0.50	0.74	0.28 ± 0.19
18	0.11	0.47	0.47	0.07	0.16	0.58	0.33 ± 0.20
19	0.01	0.37	0.35	0.00	0.14	0.54	0.47 ± 0.18
average	0.52	0.46	0.48	0.52	0.48	0.73	0.30
std	0.31	0.28	0.26	0.31	0.29	0.17	0.17

**Table 4:** Beat-to-beat detection performance analysis for diseased subjects (DS II) using the presented method compared to those metrics using the Hilbert algorithm on the same dataset.

	ICA (Fusion)				Hilbert (Accelerometer)				Hilbert (Gyroscope)			
**Subject ID**	**TPR**	**PPV**	**F1**	**RMSE**	**TPR**	**PPV**	**F1**	**RMSE**	**TPR**	**PPV**	**F1**	**RMSE**
1	0.98	0.98	0.98	42.5	0.86	0.83	0.84	114.3	0.85	0.84	0.84	118.0
2	0.93	0.93	0.93	49.6	0.98	0.99	0.99	51.6	0.99	0.99	0.99	43.5
3	0.90	0.90	0.90	63.0	0.87	0.86	0.87	44.0	0.81	0.80	0.81	154.8
4	0.94	0.95	0.94	107.5	0.87	0.88	0.88	60.9	0.84	0.84	0.84	142.6
5	0.97	0.97	0.97	33.5	0.94	0.94	0.94	51.1	0.98	0.97	0.98	36.9
6	0.81	0.79	0.80	42.2	0.45	0.41	0.43	160.9	0.71	0.62	0.66	114.0
7	0.95	0.94	0.95	39.4	0.16	0.16	0.16	94.8	0.37	0.37	0.37	71.9
8	0.92	0.92	0.92	35.5	0.83	0.67	0.75	104.1	0.85	0.67	0.75	68.1
9	0.99	0.97	0.98	44.0	0.91	0.76	0.83	251.5	0.88	0.74	0.80	255.2
10	0.99	0.99	0.99	16.9	0.98	0.98	0.98	25.5	0.99	0.99	0.99	5.6
11	0.95	0.97	0.96	38.0	0.93	0.95	0.94	75.6	0.92	0.94	0.93	76.6
12	0.98	0.70	0.81	91.2	0.99	0.77	0.87	81.1	0.99	0.77	0.87	47.3
13	0.91	0.97	0.94	57.9	0.88	0.92	0.90	30.0	0.92	0.96	0.94	33.7
14	0.99	0.99	0.99	53.7	0.93	0.93	0.93	74.3	0.92	0.93	0.92	162.7
15	0.98	0.99	0.98	16.6	0.98	0.98	0.98	31.0	0.99	0.99	0.99	20.1
16	0.76	0.82	0.79	98.2	0.55	0.50	0.52	138.1	0.69	0.61	0.65	110.0
17	0.99	0.99	0.99	24.0	0.97	0.98	0.98	66.3	0.99	0.99	0.99	11.5
18	0.98	0.98	0.98	87.7	0.99	0.99	0.99	20.4	0.99	0.99	0.99	52.3
19	0.92	0.94	0.93	168.3	0.89	0.88	0.88	125.7	0.90	0.89	0.89	87.3
average	**0.94**	**0.93**	**0.93**	**58.4**	0.84	0.81	0.82	84.3	0.87	0.84	0.85	84.9
std	0.06	0.08	0.06	37.6	0.21	0.22	0.22	56.76	0.15	0.17	0.15	62.9

**Table 5:** Correlation analysis of accelerometer-derived respiration signals (including PCA signal) against the respiration belt signal in three different breathing patterns.

Healthy Subjects (ADR and Respiration Belt)									
**Normal Paced Breathing**				**Slow Paced Breathing**			**Fast Paced Breathing**		
**Subject**	**ADRx**	**ADRy**	**PCA**	**ADRx**	**ADRy**	**PCA**	**ADRx**	**ADRy**	**PCA**
1	0.48	0.22	0.18	0.46	0.49	0.46	0.87	0.60	0.90
2	0.75	0.70	0.74	0.13	0.69	0.69	0.92	0.91	0.94
3	0.50	0.70	0.66	0.58	0.36	0.61	0.69	0.64	0.75
4	0.38	0.55	0.14	0.49	0.012	0.49	0.38	0.68	0.54
5	0.26	0.57	0.37	0.51	0.46	0.51	0.65	0.34	0.66
6	0.13	0.52	0.53	0.50	0.44	0.52	0.27	0.74	0.74
7	0.82	0.61	0.82	0.59	0.33	0.49	0.63	0.54	0.68
8	0.32	0.12	0.32	0.34	0.48	0.35	0.71	0.62	0.72
9	0.67	0.42	0.68	0.47	0.58	0.45	0.60	0.63	0.66
10	0.62	0.17	0.63	0.72	0.37	0.74	0.87	0.83	0.87
11	0.63	0.15	0.42	0.67	0.56	0.64	0.64	0.60	0.79
12	0.37	0.63	0.69	0.65	0.40	0.66	0.67	0.57	0.67
average	0.49	0.45	0.51	0.51	0.43	0.55	0.66	0.64	0.74
std	0.21	0.22	0.22	0.16	0.17	0.11	0.19	0.14	0.12
